# Injuries from electronic cigarettes, and cigarette/cigar-related paraphernalia, NEISS, 2012–2022

**DOI:** 10.1371/journal.pone.0298177

**Published:** 2024-05-24

**Authors:** R. Constance Wiener, Eric W. Lundstrom

**Affiliations:** 1 Department of Dental Public Health and Professional Practice, School of Dentistry, West Virginia University, Morgantown, West Virginia, United States of America; 2 Department of Epidemiology and Biostatistics, School of Public Health, West Virginia University, Morgantown, West Virginia, United States of America; Thamar University: Dhamar University, YEMEN

## Abstract

There is a need to determine the role of smoking/vaping related products in Emergency Department (ED) product-related injuries by age and sex to determine if interventions are warranted. These products include the combustible tobacco products’ paraphernalia to light them (CTPP), electronic nicotine delivery systems (ENDS), and electronic non-nicotine delivery system (ENNDS). Data from the National Electronic Injury Surveillance System (NEISS), years 2012–2022, were examined for injury data associated with CTPP and ENDS/ENNDS. Bivariate comparisons were conducted. There were an estimated 3,142 (95%CI: 2,384–3,975) ED-treated ENDS/ENNDS product-related injuries and 46,116 (95%CI: 38,712–53,520) CTPP product-related injuries. Males were more likely to have an ED-treated ENDS/ENNDS product-related injury than females (proportion 0.93 [95%CI: 0.82, 0.98] versus 0.70 [95%CI: 0.02, 0.19]) as well as a CTPP product-related injury than females (proportion, 0.60 [95%CI: 0.56, 0.64] versus 0.40 [95%CI: 0.37, 0.44]). There were more ED-treated ENDS/ENNDS product-related injuries among persons ≥18 years than <18 years (proportion, 0.89 [95%CI: 0.75, 0.96] versus 0.11 [95% CI: 0.4, 0.35]). There were also more ED-treated CTPP product injuries among persons ≥ 18 years than <18 years (proportion, 0.73 [95%CI: 0.68, 0.78] versus 0.27 [95%CI: 0.22, 0.32]). No change in the proportion of injuries in our sample associated with END/ENNDS over time were observed. There is a need to consider injuries related to ENDS/ENNDS and CTPP product-related injuries in the discussion of the risks associated with smoking/vaping. Although ENDS/ENNDS have had fewer ED-treated injuries, the number of such injuries has remained stable, rather than declined over the previous decade. Injury prevention is a public health imperative and targeted interventions by healthcare providers during routine care, and the use of public service announcements could specifically target adults ≥18 years. Providing peer-to-peer educational programs, and initiating similar programs targeted at males who use CTP and ENDS/ENNDS have the potential to decrease injury risk.

## Introduction

Combustible tobacco products (CTP) include cigarettes, cigars, pipes, etc. CTP’s paraphernalia (P) to light the products such as lighters, lighter fluid, etc. (all of which will be heretofore referred to as CTPP) have notorious harms and associated injuries. Similarly, electronic nicotine delivery systems (ENDS) and electronic non-nicotine delivery systems (ENNDS) have also resulted in serious harms and injuries. It is widely known that CTP are associated with cancer, lung disease, heart disease, cerebrovascular accidents, diabetes, and increases in the risk of tuberculosis, problems of the immune system, and certain eye diseases [1]. ENDS and CTP contain nicotine, a harmful, addictive toxin. The aerosol generated by ENDS/ENNDS potentially contains 98 toxicants, albeit in lower levels than cigarette smoke [2].

Additionally, both CTPP and ENDS/ENNDS have the potential for harm from fires. ENDS/ENNDS batteries have exploded, causing severe burn injuries, lacerations, fractures and other traumatic injuries [3]. Lit cigarettes, cigars, or pipes, and the process of lighting them from matches (lights), lighters, or other flame sources has also been associated with burn injuries [4]. These harms have been under-explored in the recent injury research literature, and previous researchers have indicated such burns represent an unrecognized epidemic [3].

This is particularly worrisome as, in 2023, there were 4.6% of middle school children and 10.0% of high school children who used ENDS/ENNDS [5]. Among middle school children, 5.6% of girls and 3.5% of boys currently used ENDS/ENNDS [5]. Among high school children, 12.2% of girls and 8.0% of boys currently used ENDS/ENNDS [5]. In 2023, 1.1% of middle school children and 1.9% of high school children used cigarettes in the past 30 days [5]. There were 1.1% of middle school girls and no reports of boys who currently used cigarettes [5]. There were 1.5% of high school girls and 2.3% of high school boys who currently used cigarettes [5].

Among adults, 4.5% currently used ENDS/ENNDS [6]. There were 4.0% who were female, and 5.1% who were male who currently used ENDS/ENNDS [6]. There were 14.5% adults who currently used CTP [7]. There were 11.1% of women and 18.2% of men who used CTP [7].

In one study of burns associated with ENDS/ENNDS from three burn centers in California, researchers examined reports from 2007 (when ENDS/ENNDS were introduced for sale in the U.S.) to 2016 [3]. They found 30 ENDS/ENNDS burn injuries of which 26 were related to ENDS/ENNDS battery explosions [3]. Of the 30 injuries, four were facial injuries [3]. In a retrospective case series studies of patient records from 2012–2015, 14 patients had ENDS/ENNDS burns from <1% to 6% of their total body surface area [8].

The results of a systematic review in which articles from one month (October 2019) were searched in PubMed and Embase, researchers found 20 articles with 21 study subjects who had an ENDS/ENNDS-related oromaxillofacial traumatic injury [9]. All were male. Some of the injuries included oropharyngeal burns, lacerations to the lips, tongue, soft and hard palate, face and eyes, tooth avulsion, fractures to the nose, palate, and other bones of the face [9].

National data concerning ENDS/ENNDS burns has also been used in retrospective studies. Several researchers have used the National Electronic Injury Surveillance System (NEISS) data. These reports provided descriptive accounts of the number of ENDS/ENNDS-related injuries: 2,718 from 2013 to 2017 [10]; 2,035 from 2015–2017) [11]; 2,693 from 2015–2018 [12]; 2,269 from 2015–2019 [13]; and, 1,739 in 2018 [14].

National data concerning CTPP injuries are also limited. In a 2000 study, CTP and CTPP were responsible for 100,000 U.S. fires, caused 30% of U.S. fire deaths, and resulted in $6.95 billion in fire-related costs [4]. Ignition from CTP and CTPP (smoking materials) was responsible for 50% of home upholstered furniture fire deaths from 2010–2014 [15].

The regulations relating to the prevention of injuries include the 2012 U.S. required fire standard compliant cigarettes (FSC) with bands of wrapping paper at specific intervals to reduce air flow to a cigarette that is not in use and theoretically extinguishes a cigarette not actively being smoked [16]. FSC reduced the number of fatalities in residential fires by 23%, but were not associated with a reduction in burn injuries [16]. In May 2023, the U.S. Food and Drug Administration (FDA) proposed changes in the manufacture, design, and packaging of tobacco products to improve safety and reduce injuries [17]. The proposed changes included minimizing contaminants, regulating labeling and concentrations of e-liquids, and establishing design controls [17]. Public comments continued until October 2023 and new requirements will be forthcoming [17].

Researchers using NEISS 2000–2014 data found an estimated 23,377 CTP/CTPP product-related injuries with over one-fourth (25.6%) due to running into or falling over cigarettes or lighters/fuels [18]. In another NEISS study of data from 2009–2013, the median age for CTPP (cigarette or pipe lighter) burn injuries was 13 years and 89% occurred in females [19].

There is a gap in available current data concerning ENDS/ENNDS and CTP/CTPP product-related injuries. This information is needed to inform intervention plans and policy development. Additionally, there is a gap in the current injury data for such injuries by age and sex. There is a need to determine the role of CTPP product-related injuries, and ENDS/ENNDS product-related injuries by age and sex to determine if interventions directed to a specific target group are warranted. With a previous account that female children were more likely to have cigarette product-related burn injuries, it is important to examine recent data by age and sex for current patterns. This study provides a unique contribution of updated injury monitoring data from a national perspective that has the potential to reduce ENDS/ENNDS and CTPP product-related injuries.

## Materials and methods

This research was acknowledged as non-human subject research by the West Virginia University Institutional Review Board (protocol 1910763837) as the data were anonymized, archived, and publicly available data conforming to the Declaration of Helsinki. The data were from the National Electronic Injury Surveillance System (NEISS). The NEISS data sets from 2012 to 2022 were available through the U.S. Consumer Product Safety Commission (CPSC) website [20]. NEISS data have been collected for >45 years to determine consumer product-related injuries and to improve product safety. The data are nationally representative and are based on a probability sampling of approximately 100 U.S. hospitals and hospitals in U.S. territories to represent the approximate 5,000 U.S. hospitals with emergency departments (ED) [20]. NEISS data are collected by a member of the ED team of a reporting hospital when a patient is admitted with an injury. The detailed data are recorded into the patient’s medical record. Daily, all such records are reviewed by a NEISS hospital coordinator who codes the data according to the NEISS coding manual [20]. These data are entered into a computer with a secure internet connection and are transmitted electronically to CPSC.

For this study NEISS data from years 2012–2022 were merged and examined for injuries resulting in an ED visit that were associated with CTPP or ENDS/ENNDS. NEISS codes that were extracted were: 1604 (cigarette or pipe lighters) and 0884 (batteries). Similar to previous research using NEISS data to assess ENDS/ENNDS injuries [8], the NEISS narrative field was searched using the terms “e cig,” “vape,” “cig,” “juul,” “vaping,” “nic cig,” “ette batt,” “vapor,” and “e-vig” for the ENDS/ENNDS variable. The results of the narrative field search were read and reviewed by one researcher, EWL, to ensure that each case was associated with ENDS/ENNDS batteries.

The other main variables of interest were sex (male; female) and age (≥18 years; <18 years), which were available in the data set. Also extracted from the data file were the ED treatment disposition (treated and released/examined and released without treatment; treated and transferred to another hospital; treated and admitted for hospitalization; held for observation; left without being seen/left against medical advice; fatality), location of injury (home; farm/ranch; street/highway; other public property; manufactured [mobile] home; industrial place; school; place of recreation/sports), involvement of drugs/medications (yes; no), and race/ethnicity (White; Black/African American; Asian; American Indian/Alaska Native; Native Hawaiian/Pacific Islander; other).

Data were analyzed with RStudio version 4.3.0 [21]. Bivariate analyses were conducted for differences in reported emergency department visits for CTTP and ENDS/ENNDS injuries. Two-sided t-tests were used to assess difference in mean age between CTTP and ENDS/ENNDS injuries. A significance level of <0.05 was set a priori.

## Results

There were an estimated 49,258 (95% CI = 41,284–57,232) ENDS/ENNDS and CTPP product-related injuries that resulted in ED visits. Of these, there were 3,142 (95% CI = 2,384–3,975) ENDS/ENNDS product-related injuries and 46,116 (95% CI = 38,712–53,520) CTPP product-related injuries (Table 1). Males were more likely to have an ENDS/ENNDS product-related injury requiring an ED visit than females (0.94 [95%CI: 0.83–0.98] versus 0.06 [95%CI unstable). Similarly, males were more likely to have a CTPP product-related injury than females (0.63 [95%CI: 0.59–0.66] versus 0.38 [95%CI: 0.34–0.41]).

**Table 1:** Comparison of ENDS/ENNDS and combustible tobacco product-related injuries, NEISS, 2012–2022, weighted n = 49,258 (95% CI = 41,284–57,232).

Variable	ENDS/ENNDS product-related injuries		Combustible tobacco product-related injuries		Chi Square comparison of ENDS/ENNDS and CPPT injuries
	n (95% CI)	Proportion (95% CI)	n (95% CI)	Proportion (95% CI)	p-value
Total	3142 (2384–3975)	-	46116 (38712–53520)	-	-
**Sex**					
Male	3021 (2389–3653)	0.94 (0.83–0.98)	28828 (23990–33665)	0.63 (0.59–0.66)	<0.01
Female	197 (see note)	0.06 (see note)	17288 (14108–20468)	0.38 (0.34–0.41)	
**Age**					
≥18	2890 (2166–3614)	0.90 (0.77–0.96)	33608 (27237–39979)	0.73 (0.68–0.77)	0.01
< 18	328 (see note)	0.10 (see note)	12507 (10018–14997)	0.27 (0.23–0.32)	
**Disposition**					
Treated and released, or examined and released without treatment	2108 (1478–2738)	0.68 (0.50–0.82)	37383 (31916–42849)	0.81 (0.76–0.85)	
Treated and transferred to another hospital	307 (see note)	0.09 (see note)	3348 (2289–4408)	0.07 (0.06–0.09)	0.14
Treated and admitted for hospitalization	536 (see note)	0.17 (see note)	4306 (1914–6698	0.09 (0.06–0.15)	
Held for observation	55 (see note)	0.02 (see note)	262 (see note)	0.01 (see note)	
Left without being seen/Left against medical advice	119 (see note)	0.04 (see note)	738 (see note)	0.02 (see note)	
Fatality	-		78 (see note)	<0.01 (see note)	
Not recorded	17 (see note)	<0.01 (see note)	-		
**Location of Injury**					
Home	783 (434–1132)	0.25 (0.15–0.38)	25174 (19582–30767)	0.55 (0.48–0.62)	
Farm/Ranch	-		134 (see note)	<0.01 (see note)	
Street or highway	-		850 (369–1330)	0.02 (0.01–0.03)	0.01
Other public property	283 (see note)	0.09 (see note)	2107 (1230–2984)	0.05 (0.03–0.06)	
Manufactured (mobile) home	-		-		
Industrial place	-		-		
School	-		268 (see note)	0.01 (see note)	
Place of recreation or sports	-		149 (see note)	<0.01 (see note)	
Not recorded	2076 (1327–2826)	0.66 (0.52–0.78)	17434 (13634–21234)	0.38 (0.31–0.45)	
**Drugs/Medication**					
Yes	88 (see note)	0.03 (see note)	824 (see note)	0.02 (see note)	
No	1471 (731–2211)	0.47 (0.29–0.65)	7259 (4699–9819)	0.16 (0.11–0.22)	0.28
NA	1582 (1021–2144)	0.50 (0.35–0.66)	38033 (31464–44601)	0.83 (0.77–0.87)	
**Race/ethnicity**					
White	2241 (1545–2938)	0.70 (0.54–0.82)	27162 (21410–32915)	0.59 (0.52–0.65)	
Black/African American	288 (see note)	0.09 (see note)	5368 (3621–7114)	0.12 (0.08–0.17)	0.61
Asian	16 (see note)	0.01 (see note)	63.5 (see note)	<0.01 (see note)	
American Indian/Alaska Native	-		369 (see note)	<0.01 (see note)	
Native Hawaiian/Pacific Islander	-		35 (see note)	<0.01 (see note)	
Other	64 (see note)	0.02 (see note)	2285 (1049–3521)	0.05 (0.03–0.08)	
Not Stated in ED Record	608 (see note)	0.19 (see note)	10834 (7260–14407)	0.24 (0.17–0.31)	

**Note:** NEISS indicates that estimates may be unstable due to the limited number of unweighted cases (cases < 20), limited weighted cases (<1,200), or a coefficient of variance >33%. The confidence intervals are not provided, and the unstable estimate is included for reference.

**Fig 1:**
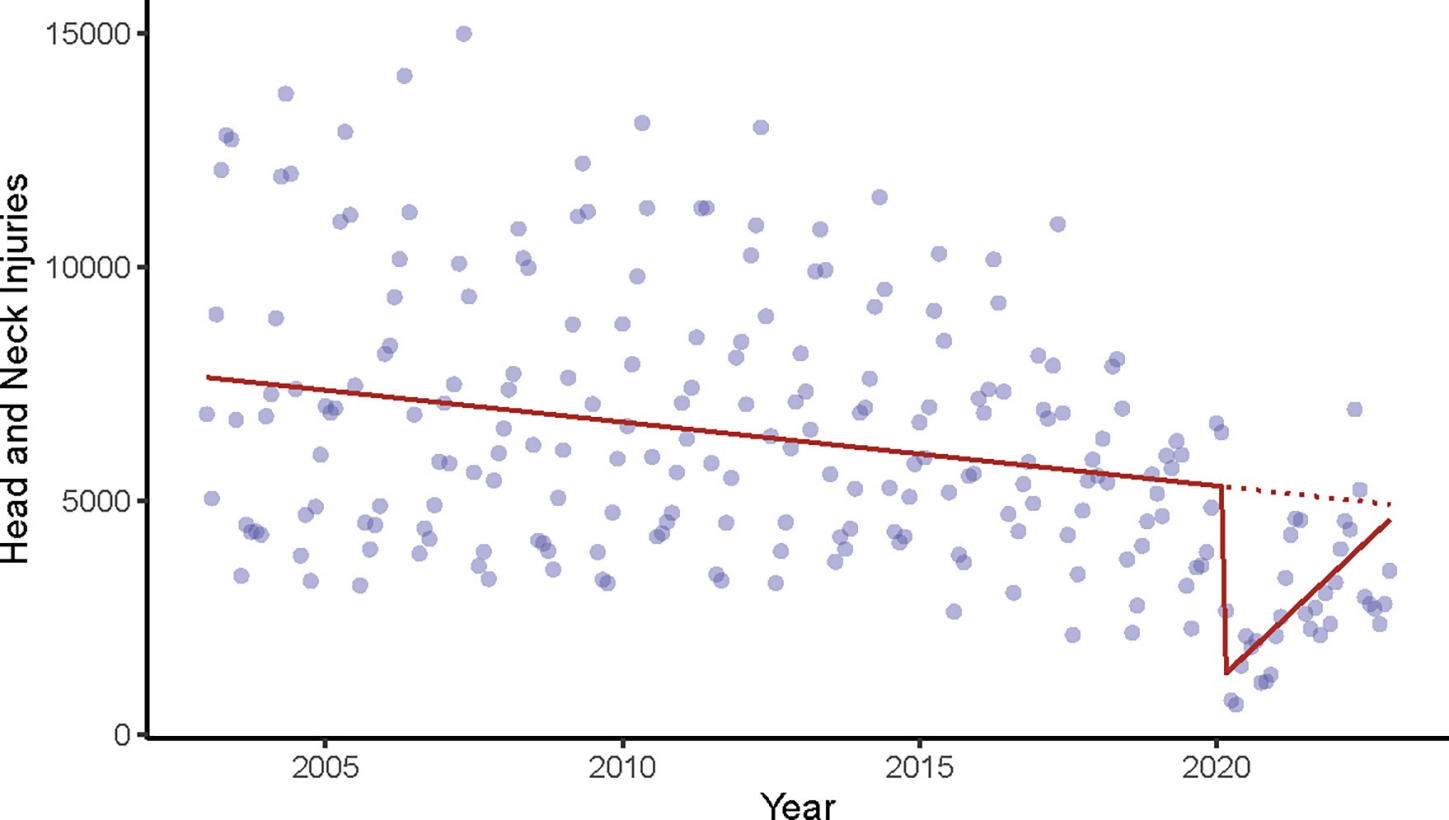
Proportion of injuries in our sample associated with END/ENNDS products, 2015–2022, weighted n = 49,258 (95% CI = 41,284–57,232). Note: Error bars denote 95% CI’s. Estimates for years 2015, 2017, 2021, and 2022 are unstable due to a coefficient of variance >33%. For these estimates, 95% CI’s are excluded and unstable estimates are shown for reference.

Mean age of ED-treated ENDS/ENNDS product-related injuries (31.0 years [95%CI: 28.1–33.9]) was significantly lower (t-test p < 0.01) than that of those sustaining CTPP injuries (38.6 years [95%CI: 36.3–40.8]). There were more ED-treated ENDS/ENNDS product-related injuries among persons ≥18 years than <18 years (0.90 [95%CI: 0.77–0.96] versus 0.10 [95% CI unstable]). There were also more ED-treated injuries associated with combustible tobacco product among persons ≥ 18 years than <18 years (proportion, 0.73 [95%CI: 0.68, 0.77] versus 0.27 [95%CI: 0.23, 0.32]). Most ENDS/ENNDS and CTPP injuries were treated and released, occurred at home (or location was not recorded), and occurred among people who were white. There were no changes in the proportion of injuries associated with END/ENNDS over time (Fig 1); no END/ENNDS injuries were captured before 2015.

## Discussion

The purpose of this study was to determine relationships between age and sex in injuries resulting in an ED visit from the paraphernalia to light combustible tobacco products and the injuries associated with ENDS/ENNDS. Adults, particularly males, ages ≥ 18 years, who use ENDS/ENNDS or CTPP, or who are in contact with them are particularly vulnerable to injuries related to ENDS/ENNDS and CTPP. In this study, there were approximately twelve times more ED-treated CTPP product-related injuries than ENDS/ENNDS product-related injuries from 2012 to 2022. Males, people ages >18 years, and people who were White were more likely to have CTPP and ENDS/ENNDS product-related injuries requiring an ED visit. Most such people who were injured were examined/treated within the ED or released without treatment.

Other researchers have found similar results as are presented in this study concerning ENDS/ENNDS injuries. In a literature review from 2015–2017, there were19 case series studies for e-cigarette burn injuries discovered in PubMed, EMBASE, and Medline [22]. Of the 90 patients in the review, 95.6% were male, and the mean age was 30.1 years [22]. Researchers of a systematic review of e-cigarette burn case studies from 2015–2017 using CINAHL, Google Scholar, and Web of Science discovered 164 injury/burn cases [23]. The cases were 90% male and most were between ages 20 to 29 years [23].

There are few studies with which to compare CTPP injuries. In this current study, a decline in CTPP injuries over the decade, 2012–2022, was not observed. In contrast, nationwide estimates are that there has been a 10% decrease in injuries from cigarette-initiated residential fires from 800 smoking injuries in 2012, to 750 smoking injuries in 2021 [24]. These estimates are not directly comparable with the current study. In our current study, the variable, CTPP injuries, included combustible tobacco paraphernalia-associated ED injuries. The CTPP variable was not specifically noted as being involved with initiating residential fires or injuries associated with residential fires in other studies.

There are several study strengths to report. The research was conducted with national data rather than case study data. The research period was ten years, providing a broad period of time to capture the characteristics of ENDS/ENNDS product expansion into the market. Both smoking and vaping products were considered in the study. The use of data from ten years of available data provided a large sample size to be sufficiently powered to address the research questions regarding differences in injuries between sexes and age groups. The NEISS data source indicated that fewer than 20 unweighted cases, or fewer than 1,200 weighted cases or a coefficient of variance greater than 33% potentially provided unstable estimates. Our sample sizes were larger than these requirements; however, 95% confidence intervals for some variables were potentially unstable. As the results are intended to be descriptive for the other variables, injury numbers were not pooled for a more complete description of the circumstances surrounding the injuries.

The study also has several limitations. First, since NEISS only captures injuries seen in U.S. EDs, this study may not be generalizable outside of the ED setting. An increasing prevalence of urgent care rather than ED use in the US may decrease the number of injuries treated in ED settings and ultimately captured by NEISS. Since our study describes a period in which sales of ENDS/ENNDS rapidly increased while awareness of their dangers was only emerging, ED and hospital record coding may not have captured all records associated with their use. This would have resulted in an underestimate of ENDS/ENNDS-related injuries in our sample. Finally, small sample sizes precluded us from reporting stable confidence intervals in several ENDS/ENNDS sub-strata. However, given the potential public health burden of ENDS/ENNDS injuries in the U.S. and a lack of scholarly information on this issue, the broad patterns observed in Table 1 may still prove useful for directing future epidemiological research and surveillance.

ENDS/ENNDS use patterns and CTP use patterns among different demographic groups are complex. Other authors have noted that the use of ENDS/ENNDS is 10% among high school children (as compared with 4.6% among middle school children [5] and 4.5% among adults [6]), with female high school students having the highest use (8.0%) [5]. However, adults have 14.5% of CTP use [7] whereas use among middle school students is 1.1% and among high school students is 1.9% [5], with adult males having the highest use (18.2%) [7]. Targeted prevention efforts to the demographic group with the greatest use/risk (adult males) may reduce the overall number of injuries.

CTPP and ENDS/ENNDS product-related injuries need to be considered in the discussion of the risks associated with smoking/vaping during smoking and vaping cessation sessions, or for educational presentations providing the rationale to not initiate the use of CTP or ENDS/ENNDS. This is particularly important in discussions with males and adults, ages ≥ 18 years. Burns and other traumatic injuries associated with these products have resulted in morbidity and mortality. Injury prevention, as well as reduction in the risk of systemic morbidity and mortality, should be considered as important topics to improve public health.

Educational initiatives are important, but also important are regulatory policies that improve the safety of potentially harmful products. As of March 2023, all states in the U.S. passed legislation prohibiting the sale of ENDS/ENNDS to minors [25]. ENDS/ENNDS have had battery fires or explosions. Regulations to improve safety features are needed such as requiring firing button locks, vent holes, and protection from overcharging. The 2024 regulations to improve safety associated with batteries include: secure compartments that require a tool or two independent hand movements to open, warning labels on packaging or the product itself, and instructions with applicable warnings [26]. Regulations are needed for compartments that keep the batteries dry and that provide adequate shielding to keep batteries from contacting metal items such as keys or coins. ENDS/ENNDS liquids have the potential for injuries related to toxic ingredients present in the liquid. Flavorings have already been banned in several states. Packaging with a list of ingredients and concentration (as required in several countries) would be helpful as would limiting the amount sold at any one purchase and expanding the number of states that ban flavorings. As of 2024, a Texas law bans packaging with cartoons, celebrities, or other youth-targeting advertising. This regulation should be considered by other state legislatures [27].

Concerning CTP, there is an FDA proposed regulation for the prohibition of menthol in CTP to address the prevention of the initiation of CTP use in youth. In 2020, the FDA required 11 cigarette package health warnings to be included on CTP. Canada is in the process of requiring manufacturers to print health warnings directly on most of the cigarettes sold in Canada by 2025. Such regulations could help to prevent CTPP injuries if required by the FDA in the U.S.

## Conclusion

Adults, particularly males, ages ≥ 18 years, who use ENDS/ENNDS and/or CTP, or who have been in contact with them, are particularly vulnerable to injuries related to ENDS/ENNDS and CTPP. Although ENDS/ENNDS have had fewer hospital-related injuries than CTPP, the number of both types of injuries has remained stable, rather than declined over the previous decade. Injury prevention is a public health imperative and targeted interventions by healthcare providers during routine care, and the use of public service announcements could specifically target adults ≥18 years. Providing peer-to-peer educational programs, and initiating similar programs targeted at males who use CTP and ENDS/ENNDS have the potential to decrease injury risk.

Data are available from:

https://www.cpsc.gov/Research—Statistics/NEISS-Injury-Data Although these data are publicly accessible, they have data use agreements required for access. The data are *described* as ED visits from January 1-December 31 in each year’s available Excel ® file. Available variables are: case number, treatment date, age, sex, race, other race, body part, diagnosis, other diagnosis, disposition, location, fire involvement, product 1, product 2, narrative, stratum, PSU, and weight. *The third party source* is the U.S. Consumer Product Safety Commission, 4330 East-West Highway, Behesda, MD 20814; 800-638-2772. If a researcher responds to the required query for access to the data, there is *no necessary contact* for use of the data [20].
